# Bacteriology and Antimicrobial Resistance in Vanuatu: January 2017 to December 2019

**DOI:** 10.3390/antibiotics9040151

**Published:** 2020-03-31

**Authors:** Nicola D. Foxlee, Nicola Townell, Mary Ann L. Tosul, Lachlan McIver, Colleen L. Lau

**Affiliations:** 1 Department of Global Health, Research School of Population Health, Australian National University, Canberra, ACT 2600, Australian; 2 Diagnostic Microbiology Development Program, Phnom Penh 12000, Cambodia; nikkitownell@hotmail.com; 3 Microbiology Department Laboratory, Vila Central Hospital, Port Vila, Vanuatu; matosul@vanuatu.gov.vu; 4 Rocketship Pacific Ltd, Port Melbourne, Melbourne 3207, Australia; lachlan@rocket-ship.org; 5 Department of Global Health, Research School of Population Health, Australian National University, Canberra 2600, Australian; colleen.lau@anu.edu.au

**Keywords:** drug resistance, antimicrobial, public health surveillance, Pacific Islands, bacteriology, epidemiology

## Abstract

The World Health Organization has identified surveillance as a key objective in the containment of antimicrobial resistance. Local antimicrobial resistance surveillance data are used to generate antibiograms to monitor resistance patterns and inform clinicians in the selection of the appropriate empiric treatment when culture results are pending, or if laboratory diagnosis is unavailable. However, producing robust bacteriology data is challenging for Pacific Island Countries and Territories with limited microbiology laboratory capacity. The aim of this study is to describe pathogen occurrence and antibiotic resistance in specimens cultured at the main referral hospital in Vanuatu. We reviewed specimen culture results for the period from January 1, 2017 to December 31, 2019. Demographic and clinical data were extracted from printed and electronic registers and described and analysed. A total of 5816 specimens were cultured, of which 21% were culture positive. *Staphylococcus* *aureus* was the predominant pathogen overall (41%), and 3% of the isolates were the methicillin-resistant *Staphylococcus aureus*. *Escherichia coli* and *Klebsiella pneumoniae* were the most frequently isolated gram-negative pathogens, of which 14% and 26% were extended-spectrum β-lactamase-producing, respectively. Our results suggest there is a need for other Pacific Island Countries and Territories to conduct similar studies. There are gaps in knowledge about antimicrobial resistance in Pacific Island Countries and Territories. Antibiograms based on reliable data will define and inform local and national actions for containing antimicrobial resistance. There is also a need to establish a regional surveillance network to strengthen national efforts and to link surveillance data for collaborative action against antimicrobial resistance.

## 1. Introduction

Addressing and containing antimicrobial resistance (AMR) is a global priority and all countries have committed to developing action plans for this purpose [1]. It is predicted that the impact of AMR on low- and lower-middle-income countries (LICs, LMICs) will be severe in terms of the health burden and economic cost of drug-resistant infections and related deaths [2]. 

**Figure 1:**
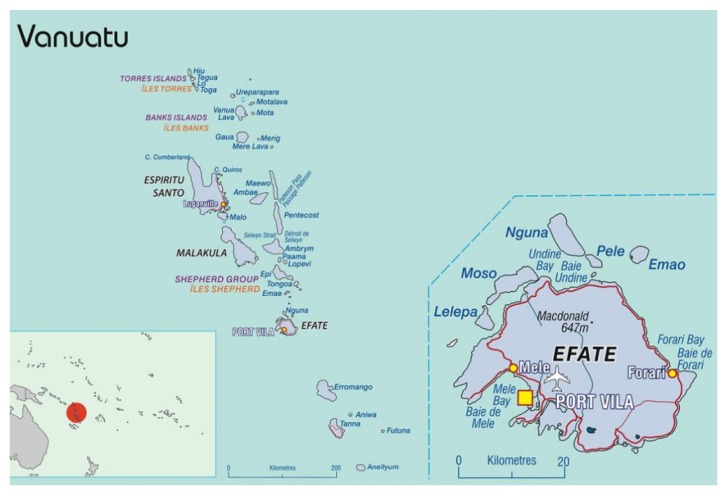
Map of Vanuatu showing Port Vila, the capital on the Island of Efate. Map was obtained with permission of the Pacific Community (SPC) http://spc.int.

Vanuatu and other Pacific Island Countries and Territories (PICTs) face challenges when it comes to combatting AMR. PICTs are in the southwest Pacific Ocean, a region prone to natural disasters and disease outbreaks, both increasingly being worsened by the effects of global environmental and climate change [3]. PICTs have small populations, relatively small land areas, limited natural resources, and economies which are supported by external aid [4]. Most PICTs are categorised as LICs or LMICs [4]. Low-income countries have the lowest indicators of socio-economic development and the lowest Human Development Index ratings in the world. They are characterised by having weaker health infrastructures, and therefore may be more susceptible to AMR (Figure 1) [2,4]. 

Good surveillance data at the local, national, and international levels are essential for understanding the global spread of AMR, and for developing policies and interventions to contain AMR [5]. In high-income countries, AMR is mapped and monitored through local and national surveillance systems, and their data are shared across global networks [6]. PICTs are not yet able to contribute to international surveillance systems, like the Global Antimicrobial Surveillance System (GLASS), developed by the World Health Organization (WHO) [1]. PICTs generally have limited laboratory capacity, often compounded by shortages of experienced staff, disrupted supplies of consumables, and limited equipment. When confronted with limited laboratory service, clinicians rely on clinical diagnosis and empirical treatment, and only request specimen tests when the first line antibiotic treatment has failed [7]. This causes laboratory data to be biased toward patients with more severe infections, for whom treatment has failed, and patients with healthcare associated infections (HCAIs). Those with milder infections, who responded to first line treatment or have community-acquired infections, are more likely to be under-diagnosed and under-reported. These factors further compromise the quality of local laboratory data. Surveillance of AMR in Vanuatu remains passive. An annual antibiogram based on the previous year’s specimen cultures is reported to hospital management and the Senior Pharmacist in the Ministry of Health [7,8]. 

Nonetheless, laboratories which function at the local level can play a crucial role in monitoring the occurrence of clinically significant organisms, assessing the prevalence of the pathogen, and determining antimicrobial susceptibility. Antibiograms, a collation of antimicrobial susceptibility results, can be used to inform health workers in selecting the most appropriate empiric treatment (whilst awaiting microbiology culture results), or when laboratory diagnosis is unavailable. Local data can contribute towards building a representative picture of national trends, and planning and measuring the impact of interventions, such as hygiene campaigns and clinical practice guidelines [5]. 

Whilst no research has been conducted on AMR in Vanuatu so far, there have been a small number of studies in other PICTs [9]. *Staphylococcus aureus* was reported as being the predominant gram-positive pathogen in the region, mainly in association with community-acquired infection. Methicillin-resistant *S. aureus* is >50% in Papua New Guinea and <20% in other PICTs [9]. Several gram-negative pathogens were reported in PICTs, including *Acinetobacter baumannii, Pseudomonas aeruginosa, Klebsiella pneumonia, and Escherichia coli,* to name a few. All were reported in association with HCAIs [9]. In other countries in WHO’s Western Pacific Region (WPR), Australia [10,11,12,13], New Zealand [14,15,16,17], and China [18], AMR is clearly a problem. Australia, New Zealand, and China have particularly close ties with Vanuatu and other PICTs through trade, investment, international aid, and seasonal workers [19,20,21]. Research into international travel and geographic trends in AMR has found that international mobility facilitates the spread of AMR [22].

In 2017, to highlight the urgent need for new treatments to be developed, WHO published a list of globally important antibiotic-resistant pathogens, for which therapeutic options are limited [23]. This list includes carbapenem-resistant *A. baumannii*, carbapenem-resistant *Enterobacteracales*, extended-spectrum β-lactamase producing (ESBL) *Enterobacteracales*, carbapenem-resistant *Pseudomonas aeruginosa* (CRPA), and MRSA. The ESCAPPM group of *Enterobacteracales* are inducible AmpC β-lactamase producing, resulting in resistance to the cephalosporins, and include the following: *Enterobacter* spp., *Serratia* spp., *Citrobacter freundi*, *Hafnia alvei*, *Acinetobacter/Aeromonas* spp., *Providencia* spp., *Proteus* spp. (not mirabilis), and *Morganella morganii* [24]. 

The aim of this study was to describe pathogen occurrence and antibiotic resistance in specimens cultured at Vila Central Hospital (VCH), the main referral hospital in Port Vila, the capital of Vanuatu, between 1 January 2017 and 31 December 2019. The results may guide selection of empiric therapy, provide data for continued guideline development, and contribute to a better understanding of AMR at the local level.

## 2. Results

### 2.1. Overview of Specimens Cultured

#### 2.1.1. Number Cultured

A total of 5,816 specimens were cultured in the microbiology laboratory between January 2017 and December 2019. This included 3084 (53%) blood specimens; 855 (15%) urine; 1216 (21%) wound/pus; 178 (3%) respiratory tract; 105 (2%) cerebrospinal fluid (CSF); 169 (3%) stool; 138 (2%) body fluids; and 71 (1%) eye, ear, and genital tract specimens (Table 1).

#### 2.1.2. Culture Results

Culture-positive pathogens were isolated from 21% (1238/5816) of the specimens and 49% (607/1238) were gram-negative. Whilst 61% (761) of the culture-positive pathogens were from pus and wound specimens, 17% (210) and 16% (194) were from blood and urine specimens, respectively. Pathogens isolated from respiratory tract, CSF, stool, body fluids, ear and eye, and genital tract specimens made up 6% (73).

The proportion of culture-positive pathogens was highest in the surgical ward with 36% (451), and this was followed by the outpatient department (OPD) with 18% (217), and the paediatric ward with 17% (213) (Table S1).

#### 2.1.3. Gender and Age Group

Overall, slightly more than 50% (625) of the pathogens were from male patients. Neonates accounted for 10% (92), child and adult patients accounted for 33% (414) and 57% (710), respectively (age data was missing for 22 pathogens overall). Details about the distribution of pathogens by type of specimen and patient age can be found in Table S1.

### 2.2. Pathogens

*S. aureus* was the most frequently isolated pathogen, accounting for 41% (502/1238) of all pathogens, followed by *E. coli*, *K. pneumoniae*, *P. aeruginosa*, and then *P*. *mirabilis*, with 15% (187), 8% (96), 6% (73), and 4% (55), respectively. The proportion of ESCAPPM pathogens cultured from all specimens was 10% (126).

#### 2.2.1. Pathogens Isolated from Blood Specimens

Whilst the total number of pathogens isolated from blood cultures was 194, half were gram-negative. The percentage of true positives and contaminants was 6.2% (194/3084) and 11.9% (368/3084), respectively. The predominant blood pathogen was *S. aureus* 23% (45), followed by *E. coli* 15% (30), *K. pneumoniae* 8% (16), and then *Streptococcus pyogenes* 7% (13). 

Overall, 24% (47) of the blood pathogens were cultured from samples taken from neonates, 34% (65) from children, and 39% (76) from adult patients (6 isolates missing age data) (Table S1).

#### 2.2.2. Pathogens Isolated from Pus and Wound Specimens

Most pathogens cultured from wound and pus specimens were gram-positive (64%; 484/761) and *S. aureus* was by far the most predominant (56%; 424) overall. The most frequently isolated gram-negative pathogen was *P. aeruginosa* (19%; 53/275), followed by *K. pneumonia* (17%; 46), *E. coli* (15%; 40), and then *Proteus mirabilis* (14%; 39). 

Just 5% (41) of the pathogens were cultured from samples taken from neonates, whilst 35% (270) and 58% (444) were taken from children and adults, respectively. Thirteen pathogens were missing age data.

#### 2.2.3. Pathogens Isolated from Urine Specimens

Ninety-four percent (94%; 198/210) of pathogens cultured from urine specimens were gram-negative. *E. coli* comprised 53% (112) and *K. pneumoniae* (12%; 26). Slightly more than half (55%; 115) of the bacteria were isolated from specimens taken from female patients.

### 2.3. Antimicrobial Susceptibility Results

**Figure 2:**
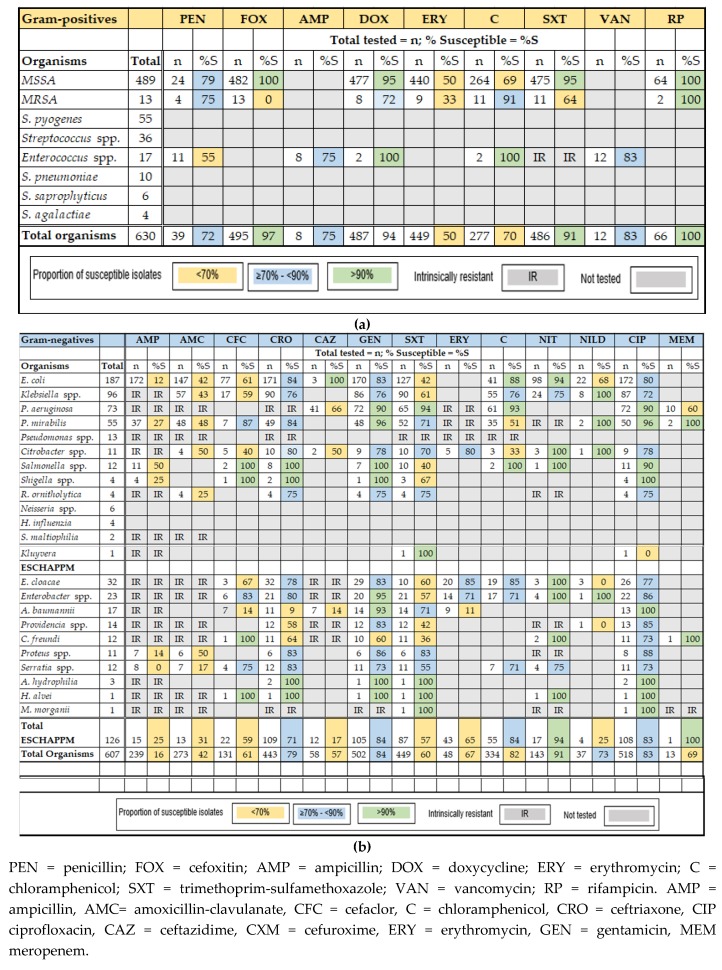
(**a**) Antibiotic susceptibility in gram-positive organisms cultured from all specimens collected at Vila Central Hospital (VCH): January 2017 to December 2019. (**b**) Antibiotic susceptibility in gram-negative organisms cultured from all specimens collected at VCH: January 2017 to December 2019.

Antibiotic susceptibility of the pathogens is profiled in a cumulative antibiogram: gram-positive (Figure 2a) and gram-negative (Figure 2b) pathogens. Antibiograms are also provided for each specimen type and classification: blood (Table S2); urine (Table S3); and wound and pus (Table S4).

Figure 2a,b is a cumulative antibiogram which profiles antibiotic susceptibility in the gram-positive and gram-negative organisms collected over the study period. Bacterial species which are intrinsically resistant to an antibiotic (antimicrobial agent) have been noted in the Table. These species have an inherent resistance to the activity of that antibiotic due to their structural and functional characteristics.

#### 2.3.1. Gram-Positive Bacteria

The most frequently isolated gram-positive organism was *S. aureus*, with a total of 502 isolates. There were 489 methicillin-susceptible *S. aureus* (MSSA) isolates and 13 MRSA. Eighty-six percent (86%) of the MSSA (423) were cultured from wound and pus specimens. Rates of susceptibility across all MSSA isolates to locally used antibiotics were >90% to cefoxitin (100%; 482/482), doxycycline (95%; 451/477), trimethoprim-sulfamethoxazole (91%; 434/475), and Rifampicin (100%; 64/64). The isolates had reduced susceptibility to chloramphenicol (69%; 183/264) and erythromycin (50%; 220/440). 

The MRSA isolates all displayed resistance to cefoxitin. Twelve of the MRSA isolates were cultured from pus specimens and one was from blood. Neonates accounted for two of the MRSA isolates, four were from children, six were collected from adults, and the patient’s age was not recorded for one isolate. 

Whilst there were other gram-positive pathogens, *Enterococcus* species (16 isolates) was the only other pathogen tested for antibiotic susceptibility, but the number tested was <12 and therefore not reported. Figure 2 and supplementary Tables S2–S4 provide additional information about antibiotic susceptibility in the gram-positive organisms.

#### 2.3.2. Gram-Negative Bacteria

*E. coli* was the predominant gram-negative pathogen and accounted for 31% (187/607) of all gram-negative organisms. The rates of susceptibility to locally used antibiotics were 12% for ampicillin (20/172), 42% for amoxicillin (62/147), 61% for cefaclor (47/77), 84% for ceftriaxone (143/171), 83% for gentamicin (141/170), 80% for ciprofloxacin (138/172), 42% for trimethoprim-sulfamethoxazole (53/127), and 90% for chloramphenicol (106/118). The susceptibility rates for nitrofurantoin and nalidixic acid (urinary isolates only) were 94% (92/98) and 68% (15/22), respectively. 

*K. pneumoniae* followed *E. coli* in frequency with 96 isolates, and these made up 16% of gram-negative organisms. The levels of susceptibility to locally used antibiotics when calculated across all specimens were: amoxicillin 43% (25/57), ceftriaxone 76% (68/90), gentamicin 76% (65/86), ciprofloxacin 72% (63/87), trimethoprim-sulfamethoxazole 61% (55/90), and chloramphenicol 76% (42/55). For urinary isolates, susceptibility to nitrofurantoin was 74% (18/24). 

All organisms in the ESCAPPM group were isolated in Vanuatu. They comprised 21% (126) of the gram-negative organisms. Levels of susceptibility to locally used antibiotics for the group were as follows: chloramphenicol 82% (45/55), ciprofloxacin 83% (90/108), ceftriaxone 77% (77/109), gentamicin 84% (88/105), trimethoprim-sulfamethoxazole 57% (50/87). More information about this group can be found in Figure 2b and Table S2–S4.

#### 2.3.3. ESBL-Producing Pathogens, Carbapenem-Resistant *P. aeruginosa* (CRPA)

At VCH, 14% (26/187) of all *E. coli* isolates and 26% (25/96) of all *Klebsiella* spp. isolates were susceptible to ceftriaxone, indicating moderate levels of ESBL. The ESBL-producing *Klebsiella* spp. was also non-susceptible to ciprofloxacin and gentamicin. Findings indicate that, whilst the total number of ESBL-producing isolates (51) identified from these two pathogens decreased from 32 (18%; 177) in 2017 to 10 (14%; 72) in 2018 and 9 (14%; 63) in 2019, there was no significant difference in the proportions being produced (p-value 0.45 (95%CI -7.02; 12.85). 

Fifty-seven percent (57%; 29) of the ESBL-producing isolates were taken from adult patients, 25% from children (13), 16% (8) from neonates, and the age of 1 patient was not recorded. 

Two *P. aeruginosa* isolates were identified in 2019 (2/10) to be CRPA: one cultured from a urine specimen from an adult patient, and the other from a pus specimen from a child patient. Ten inducible AmpC β-lactamase producing isolates, including *Enterobacter Cloacae* (5), *Citrobacter Freundii* (2), *Enterobacter* spp. (2), *and Serratia marcescens* (1) were also reported in 2019. Six were cultured from urine specimens, three from pus, and one from a blood specimen. All but two were taken from adult patients.

## 3. Discussion

This is the first study to report on pathogen occurrence and antibiotic resistance in Vanuatu. The total number of specimens cultured was 5816. Twenty-two percent (22%) of the tests yielded a positive culture, and slightly more than 50% of these were gram-positive organisms. Male patients accounted for half of the culture positive results.

*S. aureus* was the most predominant pathogen overall and was isolated from all specimen types. The proportion of MRSA was 3% (13/502). Rates of MRSA across the PICTs are highly variable [25]. MRSA was isolated from 85% (40/47) of *S. aureus* isolates collected from Papua New Guinean children (70), with hematogenous osteomyelitis in 2012 and 2017 [26] and 40% of isolates collected in 2016 in Samoa (180/428) [27]. Recent data from Cook Islands and Tonga reveal MRSA rates of 22% (129/588) and 39% (401/1051), respectively [28,29]. These rates are significantly higher than those in Vanuatu (and Solomon Islands (2%; 1/53)), [30] and it is unclear why this is so. 

To date, there has been no research about MRSA, either hospital-associated MRSA (HA-MRSA) or community-associated MRSA (CA-MRSA) in Vanuatu, and there is scarce data about HA-MRSA in other PICTs [9]. Given the strong relationships and frequent travel between PICTs, these high rates of MRSA in the region may present a significant risk of importation into Vanuatu (and Solomon Islands). Surveillance and research into the prevalence and transmission of this pathogen in all settings in Vanuatu and other PICTs is urgently needed to enable more effective monitoring and reporting. 

Apart from the MRSA isolates, several gram-negative pathogens identified on the WHO list of priority pathogens were isolated in Vanuatu. Whilst the clinical significance of these pathogens is yet to be determined, they include 26 (14%) ESBL-producing *E. coli,* 25 (26%) *K. pneumoniae* isolates and two CRPA (3%) isolates. Vanuatu has lower rates of resistance to third generation cephalosporins in *E. coli* and *K. pneumoniae* compared with other PICTs, according to previously published WHO data [25]. However, there were also 126 isolates (21% of all gram-negative isolates) belonging to the ESCAPPM group, which are inducible AmpC β-lactamase-producing, resulting in resistance to third generation cephalosporins [24]. 

Multi-drug resistant pathogens associated with HCAIs, including gram-negative *E. coli, K. pneumoniae*, *A. baumannii*, and *P. aeruginosa* have been reported in hospital settings in Fiji and the French Territories in the Pacific. During 2011 and 2012, in Fiji’s main referral hospital, 17% (114/663) of intensive care patients developed culture-confirmed sepsis. Twenty-two percent (22%; 94/437) of isolates cultured were ESBL-producing *K. pneumoniae,* whilst 21% (92/437) and 17% (73/437) were *A. baumannii* and *P. aeruginosa* [31], respectively. High rates of carbapenem-resistant *A*. *baumannii* were detected in 2004 in the Central Hospital (CH) in Noumea, (New Caledonia), and accounted for a quarter of all multi-drug resistant bacteria (24.7%; 50/202) isolated [32]. Over 4000 km away, an outbreak of carbapenem-resistant *A. baumannii* affecting 24 patients (19 colonised and 5 infected) occurred in the CH in Papeete, French Polynesia in the same year [33,34]. ESBL-producing *E. coli* and *K. pneumoniae* have also been increasing in recent years in New Zealand, although rates remain low (ESBL producing *E. coli* 2006-2008, 2.6% and 2009-2011, 3.3%) [17].

Healthcare associated infections are recognised as a problem in hospitals worldwide, resulting in increased morbidity and mortality for patients and in increased costs for the healthcare system [35]. There is insufficient data about HCAIs in low-income countries (LICs), and this may be due to the time, resources, and expertise required to conduct surveillance [35]. HCAIs are substantially higher in LICs than in middle- and high-income countries [35]. Inadequate infection prevention and control practices are common in hospitals in many LICs [36]. For these life-threatening infections, broad-spectrum therapies, such as the carbapenems, will likely be used more frequently. If resistance continues to emerge in the PICTs, these last line antibiotics which are more expensive, toxic and not readily available will be required [9].

International travel has been identified as a risk factor in the dissemination of AMR [37]. During 2016, Vanuatu received 94,463 visitors by air travel: 52% from Australia, 11% each from New Zealand and New Caledonia, 3% from China and 21% from other locations [38]. Given the levels of AMR in neighbouring islands and Asia, and the ever-increasing scale and speed of international travel amongst the populations within this group, the health systems of Vanuatu and other PICTs may struggle to contain repeated importations of multi-drug resistant bacteria into the country.

We found the rate of contaminated blood cultures was high at 12%, suggesting blood (and other) specimen collecting skills may need improvement. False-positive blood cultures (contaminated samples) lead to longer hospital stays, increased use of broad-spectrum antibiotics, exacerbating the incidence of antibiotic resistance and patient mortality, and increasing the workload for laboratory staff [39]. Findings also indicated that the uptake of laboratory services needs to be increased. Outpatient department, ER, and specialist clinics accounted for < 25% of cultures. Clinical diagnosis without quality laboratory testing may result in significant mis- or underdiagnosis, leading to poor patient outcomes [7]. Both findings point to the need to improve communication between clinicians and laboratory staff, as well as the need to establish a program of continuing laboratory education for prescribers to increase knowledge and understanding of laboratory services.

Therapeutic guidelines are evidence-informed recommendations intended to optimise patient care for use by health care providers [40]. An antibiotic prescribing guideline based on Vanuatu’s current antibiogram is being developed for Vanuatu. Once implemented, continued commitment by all stakeholders to comply with the recommendations and ensure these remain current with Vanuatu’s microbiology and susceptibility data will be imperative. The guidelines will assist clinicians in prescribing more appropriately, particularly with regard to selecting and reviewing initial empirical antibiotic treatment and reducing antibiotic consumption. This will lead to better patient outcomes and possibly the reversal of antibiotic resistance [40]. 

### Limitations of the Data

Whilst there is a small laboratory in the NPH, the laboratory has less capacity than the one at VCH, uses a paper-based record system, and has had limited internet connectivity. These factors hamper communication and data sharing. Therefore, this study describes the bacteriology and ABR in pathogens collected at Vanuatu’s referral laboratory, and it is unknown how representative this data is for the whole of Vanuatu. However, this is not uncommon in LICs, as microbiology laboratories are generally located in dense urban areas.

The data cover the years 2017 to the end of 2019. For several pathogens, there were <30 isolates identified. CLSI guidelines specify reporting only species with testing data for ≥ 30 isolates. If <30 isolates are used, CLSI recommends caution in interpreting the data, as small numbers may result in biased results. [25]. 

Slightly more than 75% of positive cultures were attributed to hospital wards. Therefore, the data is more representative of a hospital antibiogram than a community antibiogram. It is likely there is a collection bias, as specimens will be collected in patients with more severe infections, those who received prior antibiotic therapy, those whose first line treatment has failed, and those with HCAIs. Therefore, milder infections in those that responded to first line treatment, and community-acquired infections, are likely to be under-reported. This means that the profiles of pathogens and antimicrobial susceptibility are likely to bias toward a higher rate of hospital pathogens and resistance. 

## 4. Materials and Methods 

### 4.1. Location

Vanuatu, a small island nation in the southwest Pacific Ocean is made up of an archipelago of 80 islands, and 12 of these have significant populations and economy [41]. Vanuatu has a population of 300,000 [41], and is divided into six Provinces and two Health Directorates: Southern and Northern. Apart from VCH, there is another smaller referral hospital servicing the Northern Health Directorate: the Northern Provincial Hospital (NPH). These are supported by several provincial hospitals, 34 health centres, 94 dispensaries and voluntary village aid posts. VCH is primarily responsible for the Southern Provincial Health Directorate, but accepts referrals from all other hospitals. VCH’s immediate catchment, Port Vila, has a population of approximately 50,944, whilst the Province, Shefa has 96,405 [41].

VCH has 230 beds across several wards: medical, surgical, obstetrics and gynaecology (O&G), paediatrics, maternity, tuberculosis and psychiatry. There is an emergency department (ED), outpatient department (adult and children) (OPD), a radiology department, and several specialist outpatient clinics, including dental; antenatal; eye; ear, nose and throat (ENT); physiotherapy; and the diabetic foot clinic. Whilst there is a small laboratory attached to the NPH, the diagnostic laboratory in VCH is the national referral laboratory.

Currently, the microbiology laboratory documentation system is paper based, but information is transcribed into Excel spreadsheets to produce monthly statistics and annual antibiograms. An information system to support laboratory surveillance is under consideration. 

### 4.2. Quality Assurance

The VCH laboratory includes the following specialist areas: clinical biochemistry, microbiology, haematology, anatomical pathology and cytology, serology, blood bank, and tuberculosis diagnostics. The laboratory is enrolled in the External Quality Assessment Programme coordinated by the Pacific Pathology Training Centre in New Zealand. Assessments are conducted annually, and the External Quality Assessment Programme performance assessments for the microbiology laboratory in 2017, 2018, and 2019 were 90%, 95%, and 94%, respectively. 

### 4.3. Study Design

We conducted a descriptive study involving the review, collection and analysis of laboratory specimen results from January 1, 2017 to December 31, 2019. Throughout this period, the microbiology laboratory was staffed by the same qualified microbiologist, ensuring consistency in testing procedures. The following data were extracted from printed microbiology registers and Excel spreadsheets: patient number; age, gender, month, and year the specimen was received; department from which the request originated; specimen type; reasons for request; culture; and susceptibility test results. 

The analysis included the following specimen types: blood, urine, pus and wound, respiratory tract, cerebrospinal fluid (CSF), stool, body fluids, urogenital tract, ear and eye. The pus and wound specimens (abscess, lesions, ulcer, and wound) included wound discharge and pus samples. The upper and lower respiratory tract specimens included throat and nasopharyngeal swabs, sputum and, tracheal, and lung aspirate samples. Body fluid specimens included abdominal ascites, synovial, and pleural. Eye and ear samples included discharge swabs, whilst sample types from the urogenital tract included urethral and virginal swabs. Pathogen occurrence and antibiotic susceptibility are presented (Table 1 and Table S1, and Figure 2 and Tables S2–S4) respectively.

### 4.4. Microbiological Analysis

Specimens were set up on human blood agar plates and other routine plates appropriate for the specimen type. Identification of the microorganisms was conducted using conventional culture-based techniques—basic phenotypic and biochemical identification. The API 20E test kit (bioMerivex Inc. Hazelwood, MO, USA) was also used to identify and differentiate *Enterobacteracales* and other gram-negative bacteria. Identification and testing were performed according to the 2016-9 Clinical and Laboratory M100 Standards and interpretive criteria (CLSI -M100) [42]. Susceptibility testing was performed by the Kirby-Bauer disk diffusion method on Mueller Hinton agar (MHA) plates [42]. The antibiotic disks included ampicillin (10 µg), amoxicillin/clavulanate (20/10 µg), ceftazidime (30 µg), ceftriaxone (30 µg), gentamicin (10 µg), ciprofloxacin (5µg), trimethoprim/ sulfamethoxazole (1.25/23/75 µg), cefoxitin (30 µg), erythromycin (15 µg), penicillin (10 µg), nitrofurantoin (300 µg), and vancomycin (30µg) [42].

*Escherichia coli* ATCC 52922, *Staphylococcus aureus* ATCC 25923, and *Pseudomonas aeruginosa* ATCC 27853 were used as negative and positive controls, respectively, to quality control the susceptibility testing procedures in line with CLSI guidelines [42].

Extended-spectrum beta-lactamase-producing (ESBL) *E. coli* and *Klebsiella* spp. were confirmed by double disk diffusion method. The growth-inhibitory zones were measured around cefotaxime (CTX) (30 µg) and ceftazidime (CAZ) (30 µg) disks with and without clavulanic acid (CA) (10 µg). An increase of ≥5mm in the diameter of the zone of inhibition around each pair of antibiotics compared with CTX or CAZ alone, respectively, confirmed the presence of an ESBL-producing organism. MRSA were detected using a cefoxitin (30ug) disk and the isolate zone of ≤ 21 mm was confirmed phenotypically to be MRSA [42].

### 4.5. Data Analysis

For the purposes of analysis, patients were categorised as neonate (<1 month), paediatric (≥1 month to <18 years), and adult (≥18 years). The hospital wards and departments that requested the laboratory tests included: surgical, medical, O&G, paediatric, nursery, maternity, OPD, ED, dental, eye, and ENT.

Demographic and clinical data were described and analysed. For each patient, only the first isolate of a given species identified during the study period was considered, irrespective of body site (first isolate strategy) [43]. If an isolate yielded two different species, both were counted. Isolates identified as contaminants in the laboratory, those that did not grow an organism, *Bacillus* spp., and mixed bacteria were excluded from analyses. The data were entered in an Excel spreadsheet and analysed using Stata version 15 (Stata Corp, College Station, TX). Data were described and analysed as proportions. The two-sample test of proportions was used to test for difference in population proportions. A P-value of ≥ 0.05 was considered statistically significant.

### 4.6. Ethical Approval

Ethical approval was obtained from Vanuatu’s National Cultural Council, the Vanuatu Ministry of Health, and the Research Ethics Committee of the Australian National University (Protocol number 2017/056).

## 5. Conclusions

Our study characterises current pathogens and antibiotic resistance patterns in Vanuatu. There are gaps in knowledge of pathogen occurrence and AMR across PICTs. Therefore, it is important for other PICTs to conduct similar studies, so that a representative picture of pathogen occurrence and ABR can be mapped for the region. However, this will require participation in a regional surveillance network. A regional network may be facilitated by utilising the existing Pacific Pathology Training Centre network expanded to include bacterial pathogens and AMR. At least 21 laboratories across PICTs currently participate [44].

Vanuatu and other PICTs are currently working with WHO WPRO to develop action plans to address AMR following health principles. Once ratified, these plans may lead to more formal commitments to:

- Advance laboratory capacity, including the training of staff, the creation of infrastructure to improve access to microbiology services, adopting international standards, and establishing a continuing laboratory education program;
- Implement antimicrobial stewardship programmes in healthcare settings, including AMR awareness raising, improving prescribing practices, and education for infection prevention and control; and
- Develop a regional AMR surveillance network to support national containment efforts and link surveillance data to enable collaborative efforts against AMR.

## Figures and Tables

**Table 1:** Distribution of positive cultures by ward/department by specimen cultured at Vila Central Hospital (VCH): January 2017 to December 2019.

Ward or Department	Blood	Urine	Woun ^π^/Pus	RespiratoryTract *	CSF ^¥^	Stool	Body Fluids ^‡^	Ear, Eye ^±^, Oral	Urogenital Tract ^£^	Totals
n (%)										n (%)
Medicine	57 (29)	36 (17)	16 (2)	17 (49)	0	0	5 (29)	0	0	131 (11)
Paediatrics	47 (24)	18 (9)	129 (17)	7 (20)	5 (83)	1 (33)	5 (29)	1 (14)	0	213 (17)
Nursery	47 (24)	3 (1)	15 (2)	0	0	0	0	2 (29)	0	67 (5)
Surgery	14 (7)	39 (19)	389 (51)	2 (6)	1 (17)	0	4 (24)	1 (14)	1 (20)	451 (36)
Obstetrics & Gynaecology	0	8 (4)	1 (0.1)	0	0	0	2 (6)	0	0	11 (1)
Maternity	3 (2)	17 (9)	31 (4)	0	0	0	0	0	0	51 (4)
Emergency	14 (7)	18 (8)	24 (3)	1 (3)	0	1 (33)	1 (3)	0	0	59 (4)
Outpatient: adult/child	11 (6)	54(25)	143 (19)	6 (17)	0	1 (33)	0	0	2 (60)	217 (18)
Specialist Outpatient Clinic	0	9 (5)	7 (1)	2 (6)	0	0	0	3 (43)	2 (40)	23 (2)
Hospital referred	1 (1)	8 (4)	6 (1)	0	0	0	0	0	0	15 (1)
Total positive cultures	194 (100)	210 (100)	761 (100)	35 (100)	6 (100)	3 (100)	17 (100)	7 (100)	5 (100)	1238 (100)
Total lab contaminants or no growth	2890	645	455	143	99	166	121	11	48	4578
Total specimens cultured	3084	855	1216	178	105	169	138	18	53	5816

Wound ^π^ = swabs; respiratory tract * = sputum, tracheal, and lung aspirates; throat and nasopharyngeal swabs; body fluids ^‡^ = abdominal, ascites (peritoneal), joint (synovial), pleural; cerebrospinal fluid (CSF) ^¥^ = cerebrospinal fluid; ear and eye ^±^ = ear and eye discharge; urogenital ^£^ = urethral and virginal swabs.

## Supplementary Materials

The following are available online at https://www.mdpi.com/2079-6382/9/4/151/s1, Table S1: Distribution of gram-positive and gram-negative organisms isolated in the VCH laboratory: January 2017 to December 2019 by age group, Table S2(a) and (b): Antibiotic susceptibility in gram-negative and gram-positive organisms cultured from blood specimens at VCH laboratory: January 2017 to December 2019, Table S3: Antibiotic susceptibility in gram-negative organisms cultured from urine specimens at VCH laboratory: January 2017 to December 2019, Table S4(a) and (b): Antibiotic susceptibility in gram-negative and gram-positive organisms cultured from pus and wound specimens at VCH laboratory: January 2017 to December 2019

## References

[1] World Health Organization Global Action Plan on Antimicrobial Resistance. www.who.int/antimicrobial-resistance/publications/global-action-plan/en/.

[2] World Health Organization Regional Office for the Western Pacific A Primer for Media: Antimicrobial Resistance in the Western Pacific Region. iris.wpro.who.int/bitstream/handle/10665.1/13087/WPR_2016_DHS_002-007_eng.pdf.

[3] Averett N. (2016). Pacific island countries and climate change: Examining associated human health vulnerabilities. Environ. Health Perspect..

[4] United Nations Conference on Trade and Development UN Recognition of Least Developed Countries (LDC). https://unctad.org/en/Pages/ALDC/Least%20Developed%20Countries/UN-recognition-of-LDCs.aspx.

[5] World Health Organization Regional Office for the Western Pacific Antimicrobial Resistance in the Asia Pacific Region: A Development Plan. iris.wpro.who.int/bitstream/handle/10665.1/13570/9789290618126-eng.pdf.

[6] Critchley I., Karlowsky J. (2004). Optimal use of antibiotic resistance surveillance systems. Clin. Microbiol. Infect..

[7] Nkengasong J., Nsubuga P., Nwanyanwu O., Gershy-Damet G., Roscigno G., Bulterys M., Schoub B., DeCock K., Birx D. (2010). Laboratory systems and services are critical in Global Health: Time to end the neglect?. Am. J. Clin. Pathol..

[8] Ombelet S., Ronat J.B., Walsh T., Yansouni C.P., Cox J., Vlieghe E., Martiny D., Semret M., Vandenberg O., Jacobs J. (2018). Clinical bacteriology in low-resource settings: Today’s solutions. Lancet Infect. Dis..

[9] Foxlee N.D., Townell N., McIver L., Lau C.L. (2019). Antibiotic Resistance in Pacific Island Countries and Territories: A Systematic Scoping Review. Antibiotics (Basel).

[10] Australian Commission on Safety and Quality in Health Care (2019). Third Report on Antimicrobial Use and Resistance in Human Health.

[11] Coombs G., Daley D., Pearson J., Nimmo G., Collignon P., McLaws M.L., O Robinson J., Turnidge D. (2014). Community-onset of *Staphylococcus aureus* Surveillance Programme annual report, 2012. Commun. Dis. Intell..

[12] Davis J., Jones C., Cheng A., Howden B. (2019). Australia’s response to the global threat of antimicrobial resistance: Past, present and future. Med. J. Aust..

[13] Wozniak T., Paterson D., Halton K. (2017). Review of the epidemiological data regarding antimicrobial resistance in Gram-negative bacteria in Australia. Infect. Dis. Health.

[14] Blakiston M., Heffernan H., Roberts S., Freeman J. (2017). The clear and present danger of carbapenemase-producing Enterobacteriaceae (CPE) in New Zealand: Time for a national response plan. N. Z. Med. J..

[15] Heffernan H., Woodhouse R., Draper J., Ren X. 2016 Survey of Extended-Spectrum Beta-Lactamase-Producing Enterobacteriaceae. https://surv.esr.cri.nz/PDF_surveillance/Antimicrobial/ESBL/ESBL_2016.pdf.

[16] Heffernan H., Bakker S. 2017 Survey of Methicillin-Resistant Staphylococcus Aureus (MRSA). https://surv.esr.cri.nz/PDF_surveillance/Antimicrobial/MRSA/MRSA_2017.pdf.

[17] Williamson D., Baker M., French N., Thomas M. (2015). Missing in action: An antimicrobial resistance strategy for New Zealand. N. Z. Med. J..

[18] Qiao M., Ying G.G., Singer A.C., Zhu Y.G. (2018). Review of antibiotic resistance in China and its environment. Environ Int..

[19] Lau C., DXF F., Milne S., Qiu H., Sun M. (2019). Chinese venturers to Pacific Small Island Developing States: Travel and lifestyle. Int. J. Tour. Res..

[20] Curtain R., Dornan M., Howes S., Sherrell H. (2018). Pacific seasonal workers: Learning from the contrasting temporary migration outcomes in Australian and New Zealand horticulture. Asia Pac. Policy Stud..

[21] Clarke M., Feney S. (2019). The dragon versus the kangaroo: Perceptions of Chinese and Australian influence and development assistance in Vanuatu. Aust. J. Polit. Sci..

[22] Frost I., van Boeckel T.P., Pires J., Craig J., Laxminarayan R. (2019). Global geographic trends in antimicrobial resistance: The role of international travel. J. Travel Med..

[23] World Health Organization Global Priority List of Antibiotic Resistant Bacteria to Guide Research Discovery and Development of New Antibiotics. https://www.who.int/medicines/publications/WHO-PPL-Short_Summary_25Feb-ET_NM_WHO.pdf.

[24] Torok E., Moran E., Cooke F. (2017). Oxford Handbook of Infectious Diseases and Microbiology.

[25] World Health Organization Antimicrobial Resistance: Global Report on Surveillance: 2014. https://www.who.int/drugresistance/documents/surveillancereport/en/.

[26] Izzard A., Jaworski J., Drekore J., Urakoko B., Poka H., Michael A., Greenhill A. (2018). Methicillin-resistant staphylococcus aureus in Melanesian children with haematogenous osteomyelitis from the Central Highlands of Papua New Guinea. Int. J. Pediatr..

[27] Alesana-Slater J., Ritchie S., Heffernan H., Camp T., Richardson A., Herbison P., Norris P. (2011). Methicillin-resistant staphylococcus aureus: Samoa, 2007–2008. Emerg. Infect. Dis.

[28] Evert R., Cook Islands Ministry of Health (2018). Antibiotic Guidelines Cook Islands: 2018: Guidelines for Empiric and Targeted Antibiotic Treatment, Prophylaxis, Dosing and Allergies.

[29] Evert R., Tonga Ministry of Health (2018). Antibiotic Guidelines: Tonga 2018: Guidelines for Empiric and Targeted Antibiotic Treatment, Prophylaxis, Dosing and Allergies.

[30] Ferguson K., Joseph J., Kangapu S., Townell N., Duke T., Manning L., Lavu E. (2020). Quality microbiological diagnostics and antimicrobial susceptibility testing, an essential component of antimicrobial resistance surveillance and control efforts in Pacific island nations. WPSAR.

[31] Naidu K., Nabose I., Ram S., Viney K., Graham S.M., Bissell K.A. (2014). A descriptive study of nosocomial infections in an adult intensive care unit in Fiji: 2011–12. J. Trop. Med..

[32] Melot B., Colot J., Guerrier G. (2015). Bacteremic community-acquired infections due to Klebsiella pneumoniae: Clinical and microbiological presentation in New Caledonia, 2008-2013. Int. J. Infect. Dis..

[33] Le Hello S., Falcot V., Lacassin F., Baumann F., Nordmann P., Naas T. (2008). Molecular epidemiology of carbapenem-resistant Acinetobacter baumannii in New Caledonia. Clin. Microbiol. Infect..

[34] Naas T., Levy M., Hirschauer C., Marchandin H., Nordmann P. (2005). Outbreak of carbapenem-resistant Acinetobacter baumannii producing the carbapenemase OXA-23 in a tertiary care hospital of Papeete, French Polynesia. J. Clin. Microbiol..

[35] Allegranzi B., Bagheri Nejad S., Combescure C., Graafmans W., Attar H., Donaldson L., Pittet D. (2011). Burden of endemic health-care-associated infection in developing countries: Systematic review and meta-analysis. Lancet.

[36] Bardossy A.C., Bardossy A.C., Zervos J., Zervos M. (2016). Preventing Hospital-acquired Infections in Low-income and Middle-income Countries: Impact, Gaps, and Opportunities. Infect. Dis. Clin. North Am..

[37] Van der Bij A., Pitout J. (2012). The role of international travel in the worldwide spread of multiresistant Enterobacteriaceae. J. Antimicrob. Chemother..

[38] Mento S., Vanuatu National Statistics Office Impact Analysis of International Visitor Arrivals to Vanuatu: Pre and Post Cycline Pam 2015. https://vnso.gov.vu/index.php/component/advlisting/?view=download&fileId=4959.

[39] Roth A., Wiklund A., Palsson A., Melander E., Wullt M., Cronqvist J., Walder M., Sturegard E. (2010). Reducing blood culture contamination by a simple informational intervention. J. Clin. Microbiol..

[40] Kredo T., Bernhardsson S., Machingaidze S., Young T., Louw Q., Ochodo E., Grimmer K. (2016). Guide to clinical practice guidelines: The current state of play. Int. J. Q. Health Care.

[41] Vanauatu National Statistcs Office Mini Census Report: 2016 Post TC Pam. https://vnso.gov.vu/index.php/document-library.

[42] Clinical and Laboratory Standards Institute (2018). M100 Performance Standards for Antimicrobial Susceptibility Testing.

[43] Kohlmann R., Gatermann S. (2016). Analysis and presentation of cumulative antimicrobial susceptibility test data – the influence of different parameters in a routine clinical microbiology laboratory. PLoS ONE.

[44] Pacific Pathology Training Centre Pacific Pathology Training Centre Overview. pptc.org.nz/regional-external-quality-assurance-programme/.

